# Prognostic values of tissue-resident CD8^+^T cells in human hepatocellular carcinoma and intrahepatic cholangiocarcinoma

**DOI:** 10.1186/s12957-023-03009-6

**Published:** 2023-04-06

**Authors:** Lujun Chen, Hao Huang, Ziyi Huang, Junjun Chen, Yingting Liu, Yue Wu, An Li, Junwei Ge, Zhang Fang, Bin Xu, Xiao Zheng, Changping Wu

**Affiliations:** 1 Department of Tumor Biological Treatment, the Third Affiliated Hospital of Soochow University, Changzhou, 213003 Jiangsu China; 2 Jiangsu Engineering Research Center for Tumor Immunotherapy, the Third Affiliated Hospital of Soochow University, Changzhou, 213003 Jiangsu China; 3 Institute of Cell Therapy, the Third Affiliated Hospital of Soochow University, Changzhou, 213003 Jiangsu China; 4 Jiangsu Institute of Clinical Immunology, the First Affiliated Hospital of Soochow University, Suzhou, Jiangsu China; 5 Jiangsu Key Laboratory of Clinical Immunology, Soochow University, Suzhou, Jiangsu China; 6 Jiangsu Key Laboratory of Gastrointestinal Tumor Immunology, Soochow University, Suzhou, Jiangsu China

**Keywords:** Hepatocellular carcinoma, Intrahepatic Cholangiocarcinoma, Multicolor immunohistochemistry, Prognosis, Tissue-resident CD103^+^CD8^+^T cells

## Abstract

**Background:**

Tissue-resident CD8^+^T cells (CD103^+^CD8^+^T cells) are the essential effector cell population of anti-tumor immune response in tissue regional immunity. And we have reported that IL-33 can promote the proliferation and effector function of tissue-resident CD103^+^CD8^+^T cells. As of now, the immunolocalization and the prognostic values of tissue-resident CD8^+^T cells in human hepatocellular carcinoma (HCC) and intrahepatic cholangiocarcinoma (ICC) still remain to be illustrated.

**Methods:**

In our present study, we used the tissue microarrays of HCC and ICC, the multicolor immunohistochemistry (mIHC), and imaging analysis to characterize the tissue-resident CD8^+^T cells in HCC and ICC tissues. The prognostic values and clinical associations were also analyzed. We also studied the biological functions and the cell–cell communication between tumor-infiltrating CD103^+^CD8^+^T cells and other cell types in HCC and ICC based on the published single-cell RNA sequencing (scRNA-seq) data.

**Results:**

Our work unveiled the expressions of CD8 and CD103 and immunolocalization of tissue-resident CD8^+^T cells in human HCC and ICC. Elevated CD8^+^T cells indicated a better overall survival (OS) rate, implying that tumor-infiltrating CD8^+^T cells in HCC and ICC could serve as an independent prognostic factor. Moreover, the number of CD103^+^CD8^+^T cells was increased in HCC and ICC tissues compared with adjacent normal tissues. HCC patients defined as CD8^high^CD103^high^ had a better OS, and the CD8^low^CD103^low^ group tended to have a poorer prognosis in ICC. Evaluation of the CD103^+^CD8^+^T-cell ratio in CD8^+^T cells could also be a prognostic predictor for HCC and ICC patients. A higher ratio of CD103^+^CD8^+^T cells over total CD8^+^T cells in HCC tissues was negatively and significantly associated with the advanced pathological stage. The percentage of higher numbers of CD103^+^CD8^+^T cells in ICC tissues was negatively and significantly associated with the advanced pathological stage. In contrast, the higher ratio of CD103^+^CD8^+^T cells over total CD8^+^T cells in ICC tissues was negatively and significantly associated with the advanced pathological stage. In addition, single-cell transcriptomics revealed that CD103^+^CD8^+^T cells were enriched in genes associated with T-cell activation, proliferation, cytokine function, and T-cell exhaustion.

**Conclusion:**

The CD103^+^ tumor-specific T cells signified an important prognostic marker with improved OS, and the evaluation of the tissue-resident CD103^+^CD8^+^T cells might be helpful in assessing the on-treatment response of liver cancer.

**Supplementary Information:**

The online version contains supplementary material available at 10.1186/s12957-023-03009-6.

## Introduction

Hepatocellular carcinoma (HCC) and intrahepatic cholangiocarcinoma (ICC) are the two major subtypes of primary liver cancers (PLCs) [1]. The HCC accounts for up to 85%, while the ICC accounts for 10 to 15% of all PLC cases [2]. Although some early-stage PLC patients can benefit from surgical treatment and systematic anti-tumor therapies, including target therapy and immunotherapy, those with advanced stage can only receive limited benefits from few multi-kinase inhibitors [2, 3]. Currently, immunotherapeutic strategies, such as tumor vaccines, adoptive T-cell transfer therapy, immune-checkpoint inhibitors, and even immunotherapeutic strategies combined with conventional strategies, have been increasingly investigated in managing HCC and ICC. However, some are still in clinical trials [4–6]. It is worth mentioning that understanding tumor heterogeneity and the diverse landscapes of the tumor microenvironment (TME) provides a solution to unraveling how to improve the sensitivity of immunotherapies [7–10]. Novel biomarkers are identified and used to predict clinical outcome for PLCs such as surface markers, tumor mutation burden, and microsatellite instability [11–13]. Recent studies on single-cell RNA sequencing (scRNA-seq) analysis from HCC and ICC have sparked growing interest within the past few years. It has been reported that besides specific drug-resistant subpopulations, such as cancer stem cells (CSCs), the scRNA-seq can also track the development of tumor-infiltrating CD8^+^T cells. Blockade of the inhibitory molecules on the reversible exhausted CD8^+^T cells at early-stage may pave the way for developing novel immunotherapeutic strategies [14–17].

Tissue-resident CD8^+^T cells, identified as CD103^+^CD8^+^T cells, are the essential effector cell population of anti-tumor immune response in tissue regional immunity [18]. E-cadherin is the important ligand of CD103 (integrin alpha E, ITGAE) [19]. In the TME, the epithelial cancer cells can express E-cadherin, interact with CD103 on CD8^+^T cells, and then maintain the interaction of cancer cells and CD8^+^T cells, leading to the residence of tumor antigen-reactive CD8^+^T cells and the persistent anti-tumor effect in tumor tissues [20]. Results from our and other groups have confirmed that transforming growth factor-β (TGF-β) signaling or IL-33 induces CD103 expression on tumor-infiltrating CD8^+^T cells. Moreover, IL-33 can promote the proliferation and effector function of tissue-resident CD103^+^CD8^+^T cells [18, 21–23]. Furthermore, the co-expression of CD103 and CD69 on CD8^+^T cells is used to identify the distinct subset of tissue-resident memory CD8^+^T cells (T_RM_) [24, 25]. Unlike central memory and effector memory CD8^+^T cells, the CD8^+^T_RM_ cells can express unique chemokine and tissue-homing receptors but lack the lymph node homing molecules CD62L and/or CCR7, and the homing of T_RM_ cells to peripheral tissues is chemokine dependent [26, 27].

As an important surface marker of tissue-resident CD8^+^T cells, CD103 enables antigen-specific CD8^+^T cells to reside within the epithelial tissues by binding to the epithelial cell marker E-cadherin and provides first-line defense against infection in peripheral nonlymphoid tissues [24, 28, 29]. Moreover, in the TME, the tissue-resident CD103^+^CD8^+^T cells can secret granzyme B and perforin and trigger cytotoxic activities, enhancing the anti-tumor immune system response [30, 31]. We have previously reported that the higher intensity of tissue-resident CD103^+^CD8^+^T cells in human colorectal cancer tissues predicts a better overall survival (OS) of the patients [32]. Notably, the prognostic value, immunolocalization, and involvement of tissue-resident CD8^+^T cells in human HCC and ICC remain elusive. In our present study, multicolor immunohistochemistry (mIHC) and imaging analysis were used to characterize the immunolocalization of tissue-resident CD8^+^T cells in the tissue microarray (TMA) slides of human HCC and ICC. The single-cell transcriptomics analysis was performed to reveal the cellular functions and cell communications in TME of these two malignancies.

## Materials and methods

### Patients and tissue specimens

The human HCC TMA (Catalog No.: HLivH180Su11) and the human ICC TMA (Catalog No.: HIBDA160PG01) were provided by Shanghai Outdo Biotech Co., Ltd., Shanghai, China. In brief, the HCC TMA contained 94 patients (84 males and 10 females, aged from 42 to 73 years, with a median age of 53), and the survival data of all 94 patients were collected and used in the survival analysis. The detailed clinical parameters of these HCC patients are shown in Table 1. The ICC TMA contained 155 patients (96 males and 59 females, aged from 30 to 84 years, with a median age of 62), and the survival data of only 42 patients could be available and used in the survival analysis. Several cancer tissue samples (*n* = 6) were missing for the ICC TMA due to heat-induced antigen retrieval. Therefore, not all patients were involved in the final analysis of clinical parameters. The detailed clinical parameters of these patients are shown in Table 3.

**Table 1:** The correlations between CD103^+^ cells, CD8^+^T cells, and CD103^+^CD8^+^T cells in tumor tissues and clinical features of patients with HCC

Clinical parameters	Cases	Numbers of infiltrating CD103^+^ cells		*χ*^2^	*P*	Numbers of infiltrating CD8^+^T cells		*χ*^2^	*P*	Numbers of infiltrating CD103^+^CD8^+^T cells		*χ*^2^	*P*	Ratio of CD103^+^CD8^+^T/CD8^+^T cells		*χ*^2^	*P*
		Low	High			Low	High			Low	High			Low	High		
Gender																	
Male	84	38	46	0.00^a^	1.00	17	67	0.09^a^	0.76	42	42	0.74^a^	0.39	32	57	0.01^a^	0.98
Female	10	4	6			3	7			7	3			3	7		
Age (years)																	
< 57	57	24	33	0.23	0.63	12	45	0.02	0.89	31	26	0.17	0.68	19	38	1.16	0.28
≥ 57	36	17	19			8	28			18	18			16	20		
Tumor size (cm)																	
< 3.5	20	11	9	1.13	0.29	5	15	0.01^a^	0.93	11	9	0.08	0.78	5	15	1.57^a^	0.21
≥ 3.5	72	30	42			15	57			37	35			29	43		
TNM stage																	
I + II	43	21	22	2.36	0.12	8	35	0.07	0.79	23	20	0.42	0.52	14	29	0.05	0.82
III + IV	43	14	29			9	34			20	23			15	28		
T stage																	
I + II	43	21	22	2.36	0.12	8	35	0.07	0.79	23	20	0.42	0.52	14	29	0.05	0.82
III + IV	43	14	29			9	34			20	23			15	28		
Pathological stage																	
I + II	57	26	31	0.05	0.82	13	44	0.20	0.65	33	24	1.93	0.17	16	41	5.20	**0.02**
III + IV	37	16	21			7	30			16	21			19	18		

Bold signifies *P* < 0.05

^a^continuity adj. chi-square

### mIHC and imaging analysis

The mIHC was carried out by using the Opal 7-Color fluorescent IHC kit (Catalog No. NEL811001KT, PerkinElmer, USA) in combination with automated quantitative analysis (PerkinElmer, USA) according to the manufacturer’s instructions as previously described [33]. Briefly, the expressions and immunolocalization of CD103 and CD8 in the TMAs of HCC and ICC were detected to characterize tumor-infiltrating CD103^+^ cells, CD8^+^T cells, and resident CD103^+^CD8^+^T cells. In addition, cytokeratin (CK) was selected to identify the epithelial cancer cells, and the nucleus was stained by 4′,6-diamidino-2-phenylindole (DAPI). The primary antibodies, such as the monoclonal rabbit antihuman CD103 (Catalog No.: ab129202, clone: EPR4166, Abcam, USA), the monoclonal mouse antihuman CD8 (Catalog No.: M7103, clone: C8/144B, Dako, Denmark), and the anti-pan CK (Catalog No.: ab7753, clone: C11, Abcam, USA), were used in the present immunostaining. In the step of secondary antibody staining, the TMA slides were incubated with HRP-conjugated secondary antibodies (PerkinElmer, USA) in an Opal working solution (PerkinElmer, USA) and then mounted with ProLong Diamond Antifade Reagent with DAPI (Thermo Fisher, USA). Subsequently, the TissueFAXS system (TissueGnostics Asia Pacific Limited, Austria) was selected to perform the panoramic multispectral scanning of TMA slides. The acquired images were processed using StrataQuest analysis software (Version No. 7.0.1.165, TissueGnostics Asia Pacific Limited, Austria) as previously described [33]. In our present study, the DAPI staining was selected to generate a binary mask of all viable cells in the image, and then, the numbers of CD103^+^ cells, CD8^+^T cells, and CD103^+^CD8^+^T cells were counted and recorded. Finally, the ratio of CD103^+^ cells over total DAPI-stained cells, the ratio of CD8^+^T cells over total DAPI-stained cells, the ratio of tissue-resident CD103^+^CD8^+^T cells over total DAPI-stained cells, and the ratio of tissue-resident CD103^+^CD8^+^T cells over total CD8^+^T cells were involved in the correlation analysis of between clinical parameters and patients’ survival.

### scRNA-seq data analysis

scRNA-seq data of CD3^+^TILs in HCC, total tumors in ICC, and total tumor tissues in HCC combined with ICC were collected from the Gene Expression Omnibus (GEO) database [14, 17]. All analysis and visualizations were carried out using R-3.6.3 software. Seurat (Version: 3.2.3) was used to process scRNA-seq data following the official standard process. In brief, count matrix downloaded from GEO datasets was used to create Seurat object by CreateSeuratObject function and normalized by NormalizeData function. Then, top 2000 highly variable genes were selected by FindVariableFeatures function. Immediately, all genes were centered by ScaleData function. For dimensionality reduction and clustering analysis, RunPCA function was applied to run principal component analysis (PCA), and RunUMAP function using top 20 principal components (PCs) was applied to run the Uniform Manifold Approximation and Projection (UMAP) dimensional reduction. In addition, the batch effect between different samples was removed by the harmony package (Version: 0.1.0). Subsequently, CD8^+^TIL subsets were extracted using marker genes *CD3E* and *CD8A*. Dimplot and Featureplot were used to visualize the results.

### Correlation analysis

The Rmagic package (Version: 2.0.3) was used to de-noise the cell count matrix and fill in missing transcripts to address technical noise in single-cell data. Subsequently, the imputation data were used to obscure meaningful gene–gene relationships between *ITGAE* and selected immune genes. The correlation heatmap was plotted by the Pheatmap package (Version: 1.0.12) using selected immune genes whose correlation coefficient with CD103 was more than 0.3 (*r* > 0.3).

### Gene set enrichment analysis

Immune genes used for the correlation heatmap were split into three groups. Briefly, the *cutree* function was used to split the gene cluster tree, and “ntree” was set as 3 to split selected genes into three groups. Subsequently, genes clustered into the group containing *ITGAE* were used for GO enrichment analysis. All enrichment analysis plots were visualized by the *cneplot* function packaged by ClusterProfiler (Version: 3.14.3).

### Cell–cell communication analysis

Signature genes of CD103^+^CD8^+^TILs from HCC and ICC were merged as the CD103^+^CD8^+^TIL gene set. Briefly, scRNA-seq data containing HCC and ICC were processed following the official standard process [7]. Then, each cell was annotated following the article labeled. Next, the impact of other cellular components in TME was analyzed through NicheNetr (Version: 1.0.0). All visualization functions were packaged into the NicheNetr package.

### Statistical analyses

Statistical analysis was conducted using the unpaired Student’s *t*-test, two-way ANOVA, or the log-rank survival analysis. Survival (Version: 3.3–1) and survminer (Version: 0.4.9) packages were used for survival analysis. The association between CD8^+^T cells, CD103^+^ cells, and CD103^+^CD8^+^ cells and clinical parameters was evaluated by chi-squared test. Univariate and multivariate analyses were performed by Cox proportional-hazards regression, and hazard ratio and 95% confidence intervals were reported. A *P*-value of < 0.05 was considered statistically significant.

## Results

### Expression of CD103 and immunolocalization of tissue-resident CD8^+^T cells in human HCC and ICC tissues

**Fig. 1:**
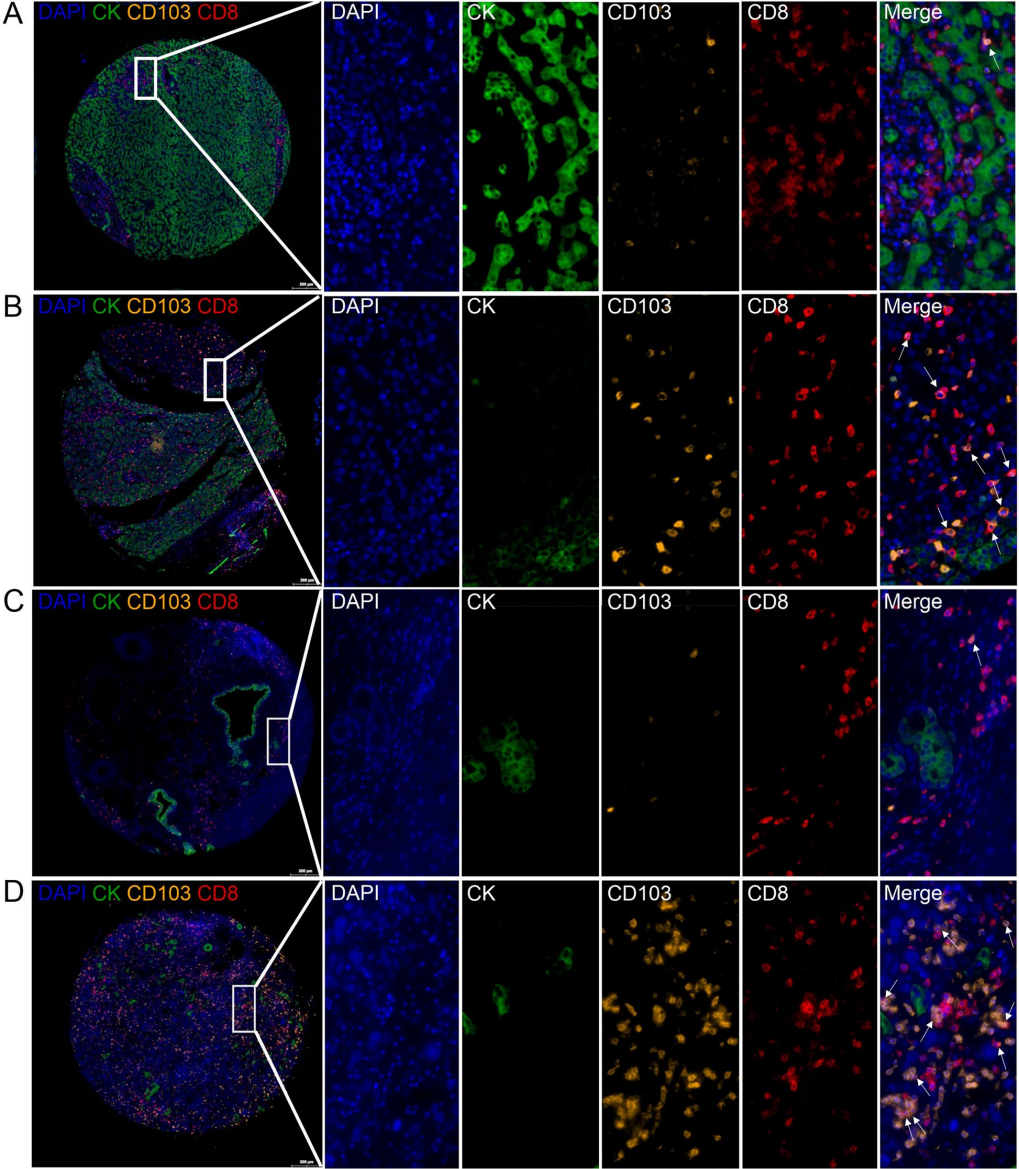
Characterization of the expressions and localizations of CD8 and CD103 in human HCC and ICC tissues by mIHC. An enlarged subsection of the core was highlighted, showing each of the individual markers in the composite image after spectral unmixing, together with the DAPI nuclear marker (pseudo-colored blue) and the autofluorescence signal (pseudo-colored black). **A** The expressions of CD8 (membrane, pseudo-colored red) and CD103 (membrane, pseudo-colored brown) in adjacent normal tissues of HCC identified by CK (membrane, pseudo-colored green), together with the DAPI nuclear marker (pseudo-colored blue). **B** Expressions of CD8 and CD103 in tumor tissues of HCC. **C** Expressions of CD8 and CD103 in adjacent normal tissues of ICC. **D** Expressions of CD8 and CD103 in tumor tissues of ICC. Scale bar for the first image of A, B, C, or D, 200 μm

In our present study, we aimed to examine the expression of CD103 and immunolocalization of tissue-resident CD8^+^T cells in human HCC and ICC tissues and to analyze their clinical associations with the patients. Based on the mIHC and imaging analysis (Fig. 1), the membranous staining of CD103 could be found on infiltrating immune cells. Besides, the membranous staining of CD8 could be found on infiltrating CD8^+^T cells. Then, the tissue-resident CD8^+^T cells, which were defined as CD103^+^CD8^+^T cells, could be found in adjacent normal tissues (Fig. 1A and C, adjacent normal tissues for HCC and ICC, respectively) and carcinoma tissues (Fig. 1B and D, HCC and ICC tissues, respectively). Supplementary Fig. 1A shows that the percentage of infiltrating CD8^+^T cells in human HCC tissues was significantly higher compared with the adjacent normal tissues (*P* = 0.0024). The percentage of infiltrating CD103^+^ immune cells in human HCC tissues was significantly higher compared with the adjacent normal tissues (*P* = 0.0307, Supplementary Fig. 1B), while no difference was found about the percentage of infiltrating tissue-resident CD103^+^CD8^+^T cells (*P* = 0.681, Supplementary Fig. 1C). Moreover, we neither found any differences between adjacent normal tissues and ICC tissues regarding the percentage of infiltrating CD103^+^ immune cells (*P* = 0.669, Supplementary Fig. 1D), the percentage of infiltrating CD8^+^T cells (*P* = 0.452, Supplementary Fig. 1E), nor the percentage of tissue-resident CD103^+^CD8^+^T cells (*P* = 0.668, Supplementary Fig. 1F). We needed to mention that during the heat-induced antigen retrieval, 6 cases of the cancer tissues of the ICC TMA were missed, and all the other cases were involved in the statistical analysis.

### Prognostic values of tumor-infiltrating CD8^+^T cells, CD103^+^ immune cells, and tissue-resident CD103^+^CD8^+^T cells in human HCC and ICC tissues

**Fig. 2:**
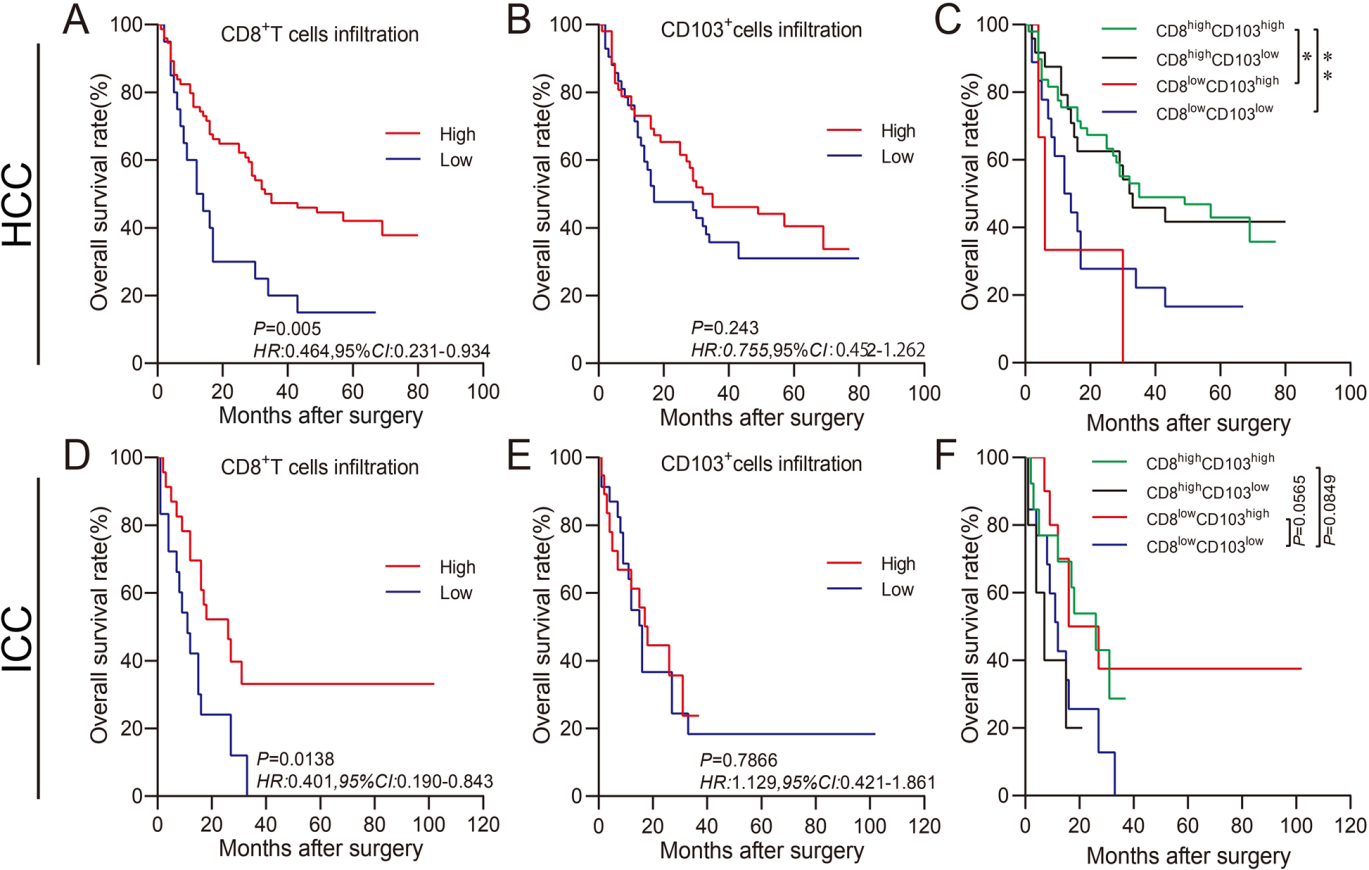
Prognostic values of CD8 and CD103 expressions in tumor tissues for the patients with HCC or ICC. Kaplan–Meier survival analysis was performed to predict the prognostic values of tumor-infiltrating CD8^+^T cells and CD103^+^ immune cells in human HCC or ICC tissues. **A**, **B**, **D**, and **E** Patients were stratified according to the infiltration intensities of tumor-infiltrating CD8^+^T cells or CD103^+^ immune cells (high infiltration in red and low infiltration in blue). **C** and **F** Patients were categorized into four subpopulations as CD8^high^ CD103^high^ (green), CD8^high^ CD103^low^ (black), CD8^low^ CD103^high^ (red), and CD8^low^ CD103^low^ (blue). Cutoffs for low and high expressions were defined with the Cutoff Finder method. *P < 0.05, **P < 0.01

**Fig. 3:**
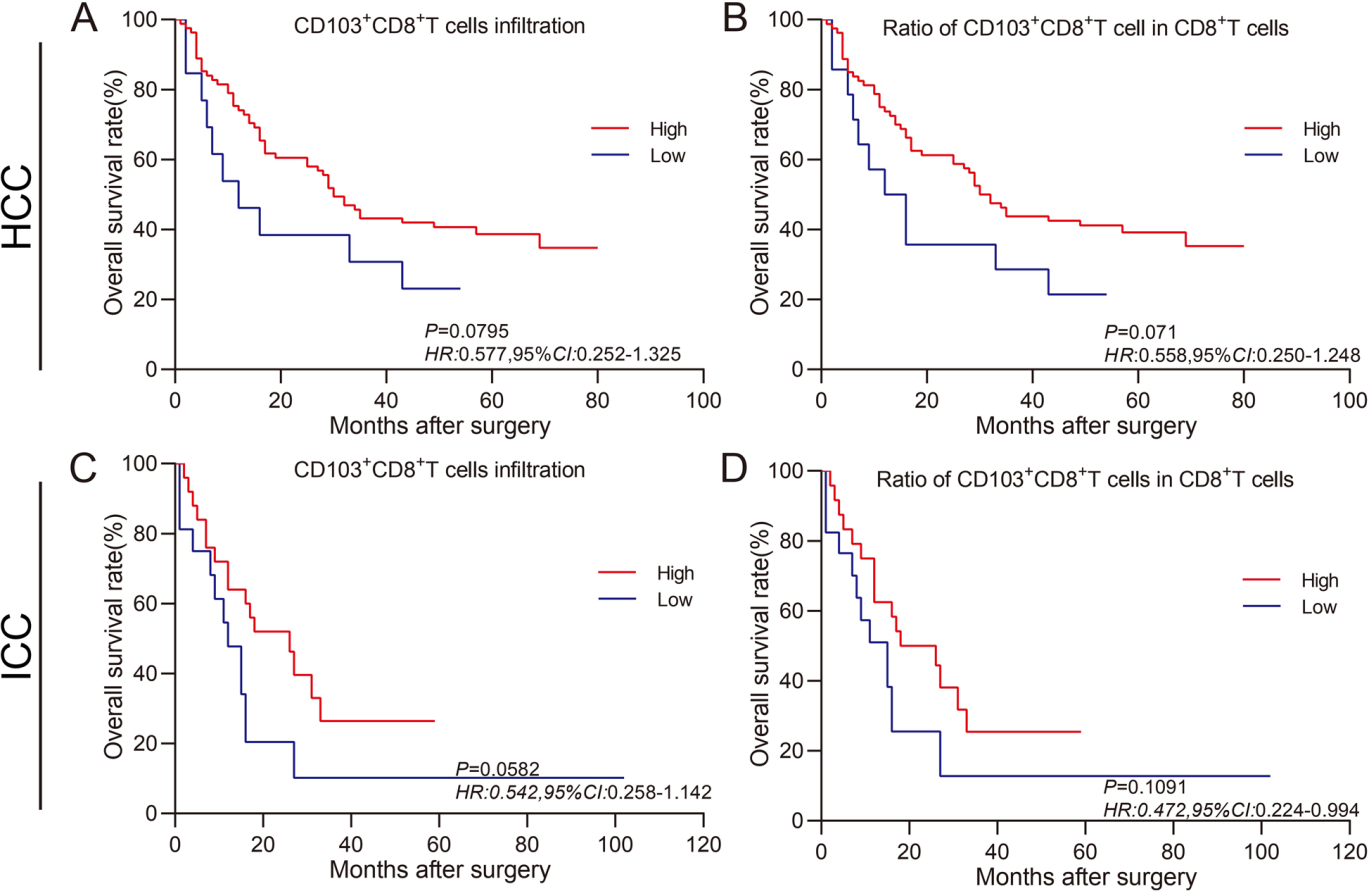
Prognostic values of tissue-resident CD103^+^CD8^+^T cells in tumor tissues for the patients with HCC or ICC. Kaplan–Meier survival analysis was performed to predict the prognostic values of tumor-infiltrating tissue-resident CD103^+^CD8^+^T cells in human HCC or ICC tissues. **A** and **C** Patients with high intensity of tissue-resident CD103^+^CD8^+^T cells (red) showed a better OS rate than those with low intensity of tissue-resident CD103^+^CD8^+^T cells (blue), *P* = 0.0795 and *P* = 0.0582 in HCC and ICC, respectively. **B** and **D** Patients with a high ratio of CD103^+^CD8^+^T cells over CD8^+^T cells (red) showed a better OS rate than those with a low ratio of CD103^+^CD8^+^T cells in CD8^+^T cells (blue), *P* = 0.071 and *P* = 0.1091 in HCC and ICC, respectively

In our present study, we also aimed to figure out the prognostic values of tumor-infiltrating CD8^+^T cells, CD103^+^ immune cells, and tissue-resident CD103^+^CD8^+^T cells in human HCC and ICC tissues. Figure 2A shows that in human HCC, the patients with higher infiltration of CD8^+^T cells showed a better OS than those with lower infiltration of CD8^+^T cells (*P* = 0.005, *HR* = 0.464, 95% *CI*: 0.231–0.934). In addition, the OS of the patients with higher infiltration of CD103^+^ immune cells was increased compared with those with lower infiltration of CD103^+^ immune cells (*P* = 0.243, *HR* = 0.755, 95% *CI*: 0.452–1.262, Fig. 2B). Figure 2C shows that in human HCC, the CD8^high^CD103^high^ patients exhibited an improved OS compared with CD8^low^CD103^high^ or CD8^low^CD103^low^ patients (*P* < 0.05 and *P* < 0.01, respectively). Figure 3A shows that the OS rate of ICC patients with higher infiltration of CD8^+^T cells was significantly prolonged than those with lower infiltration of CD8^+^T cells (*P* = 0.0138, *HR* = 0.401, 95% *CI*: 0.190–0.843). There was no significant difference in OS between the patients with higher infiltration of CD103^+^ immune cells and those with lower infiltration of CD103^+^ immune cells (*P* = 0.7866, *HR* = 1.129, 95% *CI*: 0.421–1.861, Fig. 2B). Figure 2C reveals that in human ICC, the OS of the patients with CD8^low^CD103^low^ expression was poorer than that of patients with CD8^high^CD103^high^ (*P* = 0.0849) or CD8^low^CD103^high^ expression (*P* = 0.0565).

Next, we noticed that in human HCC, the OS of the patients with a higher proportion of CD103^+^CD8^+^T cells was better than that of patients with a lower proportion of CD103^+^CD8^+^T cells (*P* = 0.0795, *HR* = 0.577, 95% *CI*: 0.252–1.325, Fig. 3A). Furthermore, patients with a higher ratio of CD103^+^CD8^+^T cells over total CD8^+^T cells had a better OS than those with a lower ratio (*P* = 0.071, *HR* = 0.558, 95% *CI*: 0.250–1.248, Fig. 3B). Furthermore, in human ICC, patients with a higher proportion of CD103^+^CD8^+^T cells lived longer than patients with a lower proportion of CD103^+^CD8^+^T cells (*P* = 0.0582, *HR* = 0.542, 95% *CI*: 0.258–1.142, Fig. 3C), and patients with a higher ratio of CD103^+^CD8^+^T cells over total CD8^+^T cells displayed a better OS than those with a lower ratio of CD103^+^CD8^+^T cells over total CD8^+^T cells (*P* = 0.1091, *HR* = 0.472, 95% *CI*: 0.224–0.994, Fig. 3B).

### Correlations between patients’ clinical parameters and the intensities of tumor-infiltrating CD8^+^T cells, CD103^+^ immune cells, and tissue-resident CD103CD8^+^T cells in human HCC and ICC tissues 

In our present study, we also aimed to investigate the correlations between patients’ clinical parameters and the intensities of tumor-infiltrating CD8^+^T cells, CD103^+^ immune cells, and tissue-resident CD103^+^CD8^+^T cells in human HCC and ICC tissues. Table 1 shows that a higher ratio of CD103^+^CD8^+^T cells over total CD8^+^T cells in HCC tissues was negatively and significantly associated with the advanced pathological stage (cut-off value = 0.01, *χ*^2^ = 5.20, *P* = 0.02). Furthermore, Table 2 reveals that the intensity of infiltrating CD8^+^T cells in HCC tissues could be an independent prognostic factor for the survival prediction of HCC patients (uni-variate: *HR* = 0.451, 95% *CI*: 0.255–0.798, *P* = 0.006, multi-variate: *HR* = 0.470, 95% *CI*: 0.238–0.928, *P* = 0.030).

**Table 2:** Univariate and multivariate analysis of clinical parameters of patients with HCC

Clinical parameters	Uni-variate		Multi-variate	
	*HR (95% CI)*	*P*-value	*HR (95% CI)*	*P*-value
Pathological stage ((III + IV)/(I + II))	1.403 (0.194–10.14)	0.737	1.093 (0.143–8.264)	0.932
Intensity of infiltrating CD8^+^T cells (high/low)	0.451 (0.255–0.798)	**0.006**	0.470 (0.238–0.928)	**0.030**
Intensity of infiltrating CD103^+^ cells (high/low)	0.754 (0.454–1.252)	0.275	1.067 (0.576–1.978)	0.826
Intensity of infiltrating CD103^+^CD8^+^T cells (high/low)	0.572 (0.289–1.133)	0.109	0.791 (0.098–6.415)	0.357
Ratio of CD103^+^CD8^+^T cells over total CD8^+^T cells (high/low)	0.558 (0.250–1.247)	0.071	1.200 (1.523–9.441)	0.862

Bold signifies *P* < 0.05

Table 3 indicates that higher numbers of CD103^+^CD8^+^T cells in human ICC tissues were negatively and significantly associated with the tumor size (*χ*^2^ = 11.99, *P* < 0.001). We also found that a higher ratio of CD103^+^CD8^+^T cells over total CD8^+^T cells in ICC tissues was negatively and significantly associated with the advanced pathological stage (cut-off value = 0.06, *χ*^2^ = 10.22, *P* = 0.001). Table 4 shows that a higher intensity of infiltrating CD8^+^T cells in ICC tissues (uni-variate: *HR* = 0.401, 95% *CI*: 0.190–0.843, *P* = 0.016) and infiltrating CD103^+^CD8^+^T cells in ICC tissues (uni-variate: *HR* = 0.472, 95% *CI*: 0.224–0.994, *P* = 0.050) could predict a better survival of the ICC patients.

**Table 3:** The correlation between CD103^+^ cells, CD8^+^T cells, and CD103^+^CD8^+^T cells in tumor tissues and clinical features of patients with ICC

Clinical parameters	Cases	Numbers of infiltrating CD103^+^ cells		*χ*^2^	*P*	Numbers of infiltrating CD8^+^T cells		*χ*^2^	*P*	Numbers of infiltrating CD103^+^CD8^+^T cells		*χ*^2^	*P*	Ratio of CD103^+^CD8^+^T/CD8^+^T cells		*χ*^2^	*P*
		Low	High			Low	High			Low	High			Low	High		
Gender																	
Male	92	42	50	1.07	0.30	39	53	1.02	0.31	28	64	0.35	0.56	28	64	2.77	1.00
Female	57	31	26			29	28			20	37			25	32		
Age (years)																	
< 62	71	34	37	0.03	0.86	30	41	0.61	0.43	20	51	0.81	0.37	22	49	1.03	0.31
≥ 62	77	38	39			37	40			27	50			30	47		
Tumor size (cm)																	
< 5	38	12	26	3.57	0.06	13	25	2.27	0.13	2	36	11.99^a^	**< 0.001**	11	27	0.67	0.41
≥ 5	99	49	50			48	51			34	65			36	63		
T stage																	
T_1_ + T_2_	29	16	13	1.28^a^	0.26	15	14	0.22^a^	0.64	12	17	0.007^a^	0.93	12	17	0.007^a^	0.93
T_3_ + T_4_	4	1	3			1	3			1	3			1	3		
N stage																	
N_0_	43	18	25	0.01	0.94	18	25	0.01	0.94	13	30	0.25	0.62	19	24	0.37	0.55
N_1_ + N_2_	22	9	13			9	13			8	14			8	14		
M stage																	
M_0_	143	69	74	0.22^a^	0.64	62	79	1.20^a^	0.27	45	98	0.256^a^	0.61	45	98	0.00^a^	1.00
M_1_	6	4	2			4	2			3	3			2	4		
Pathological stage																	
I + II	83	40	43	0.02	0.90	36	47	0.27	0.60	26	57	0.02	0.90	10	73	10.22	**0.001**
III + IV	65	32	33			31	34			21	44			22	43		

Bold signifies *P* < 0.05

^a^continuity adj. chi-square

**Table 4:** Univariate and multivariate analysis of clinical parameters of patients with ICC

Clinical parameters	Uni-variate		Multi-variate	
	*HR (95% CI)*	*P*-value	*HR (95% CI)*	*P*-value
Pathological stage ((III + IV)/(I + II))	0.704 (0.332–1.491)	0.359	0.978 (0.424–2.258)	0.958
Intensity of infiltrating CD8^+^T cells (high/low)	0.401 (0.190–0.843)	**0.016**	0.403 (0.149–1.086)	0.072
Intensity of infiltrating CD103^+^ cells (high/low)	1.129 (0.421–1.861)	0.786	1.559 (0.607–4.005)	0.357
Intensity of infiltrating CD103^+^CD8^+^T cells (high/low)	0.472 (0.224–0.994)	**0.050**	0.452 (0.140–1.462)	0.185
Ratio of CD103^+^CD8^+^T cells over total CD8^+^T cells (high/low)	0.542 (0.258–1.142)	0.107	1.424 (0.468–4.336)	0.533

Bold signifies *P* < 0.05

### The role of CD103 in CD8^+^TILs in human HCC

**Fig. 4:**
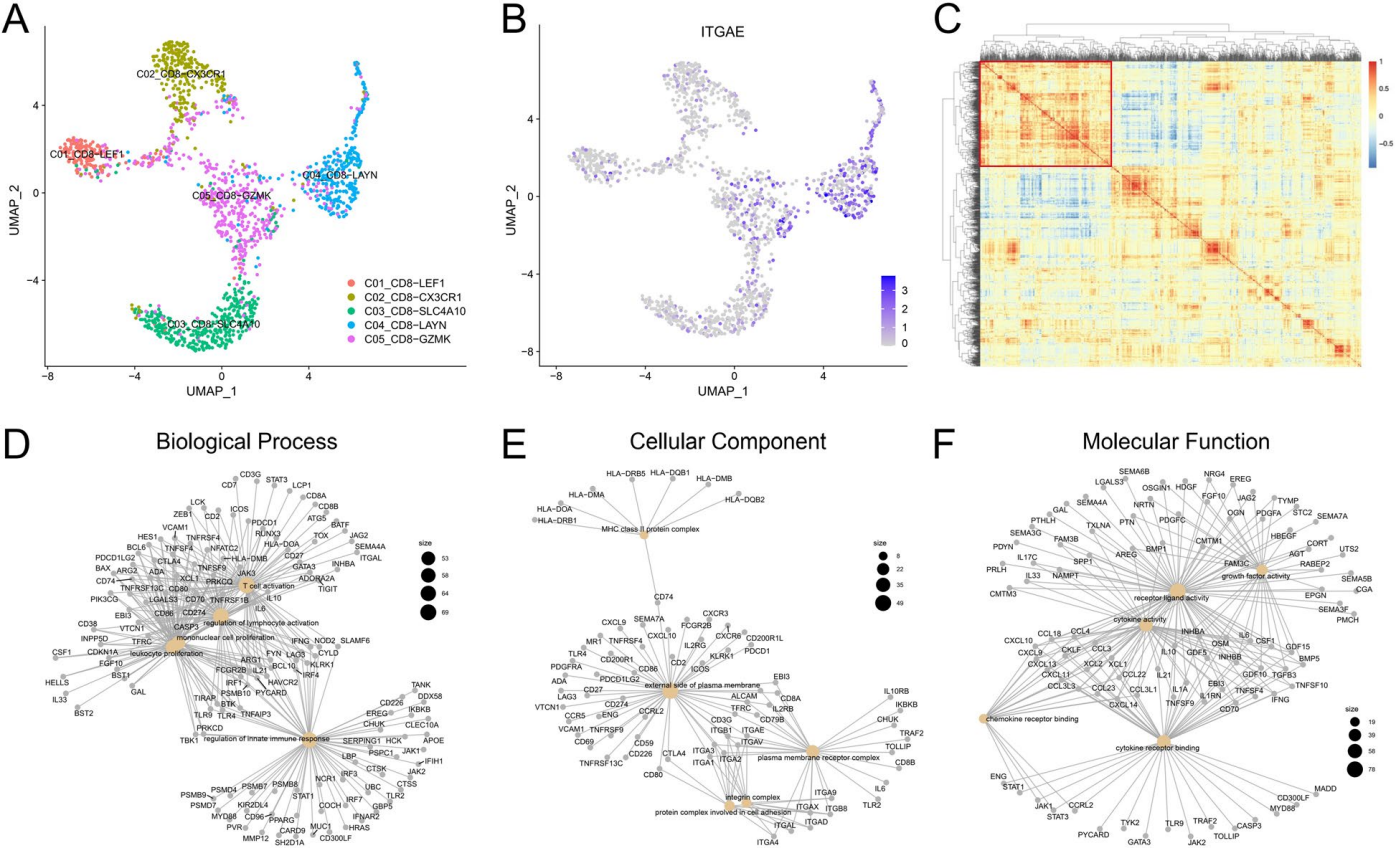
Identification and characterization of CD103^+^CD8^+^T cells in HCC. **A** UMAP plot of sub-clustered CD103^+^CD8^+^T cells labeled with cell annotations provided by the article [14]. **B** UMAP showed *ITGAE* expression in CD8^+^TILs. **C** Heatmap showed correlations between *ITGAE* and other immune-related genes in CD103^+^CD8^+^TILs. **D**, **E**, and **F** GO enrichment analysis of *ITGAE*-related immune genes (clustered with *ITGAE* in C), and three network plots showed biological process, cellular component, and molecular function

scRNA-seq data of CD3^+^TILs in HCC were collected from the GEO datasets (GSE: 98638) [14]. Seurat package was used for the following analysis. UMAP reduction result of the CD8^+^TIL subset was presented according to the article (Fig. 4A). The expression of CD103 (gene: *ITGAE*) in CD8^+^TILs showed that CD103^+^CD8^+^TILs highly expressed *LAYN*, which is one of the critical exhaustion-related genes (Fig. 4B). *LAYN* expression potentially contributed to the suppressive function of tumor-infiltrating exhausted CD8^+^T cells and Tregs [34]. To further explore how CD8^+^CD103^+^TILs exerted regulatory functions, we performed correlation analysis on genes involved in immune responses of CD8^+^TILs. We showed that the selected immune-related genes could be classified into three types, and annotated *ITGAE* had strong correlations among immune genes (Fig. 4C, highlighted by the red box). Next, genes with a high correlation with *ITGAE* were selected for Gene Ontology (GO) analysis (Fig. 4D to F). Biological processes demonstrated that the expression of *ITGAE* in CD8^+^T cells was correlated with the activation and proliferation of T cells (Fig. 4D). Cellular component analysis showed that *ITGAE* was involved in antigen processing and presentation of MHC molecules, as well as the expressions of membrane receptors and integrin-related molecules (Fig. 4E). Moreover, molecular function analysis showed that CD103 was closely related to the production of cytokines, chemokines, and growth factors (Fig. 4F).

### The role of CD103 in CD8^+^TILs in human ICC

**Fig. 5:**
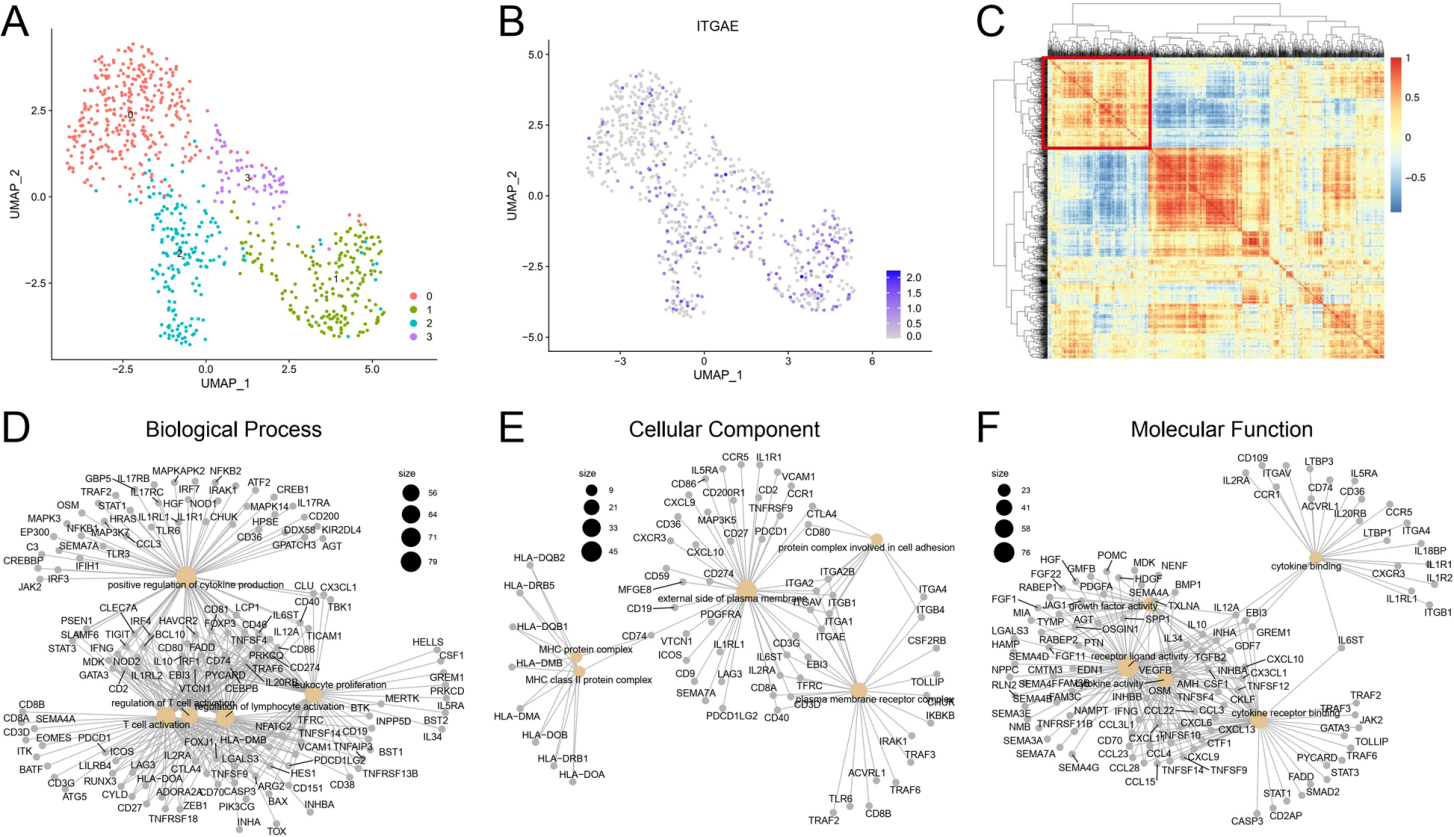
Identification and characterization of CD103^+^CD8^+^T cells in ICC. **A** UMAP plot of scRNA-seq data colored by Seurat cluster. **B** UMAP showed *ITGAE* expression in CD8^+^TILs. **C** Heatmap showed correlations between *ITGAE* and other immune-related genes in CD103^+^CD8^+^TILs. **D**, **E**, and **F** GO enrichment analysis of CD103-related immune genes (clustered with CD103 in C) and three network plots showed biological process, cellular component, and molecular function

scRNA-seq data of CD3^+^TILs in ICC were collected from GEO datasets (GSE: 138709) [17]. Analysis was performed as mentioned above (Seurat 3.0 and UMAP) (Fig. 5A). Similarly, the expression of *ITGAE* in each CD8^+^TILs set was observed, suggesting that these cells were exhausted CD8^+^T cells (data not shown) (Fig. 5B). All selected immune genes in CD8^+^TILs were clustered into three groups, and *ITGAE* significantly correlated with other immune genes, which represented that it may be an important immune regulatory gene (Fig. 5C, highlighted by the red box). *ITGAE* in ICC played an important role in antigen presentation, T-cell activation, and proliferation (Fig. 5D to F).

### Cell–cell communication between CD103^+^CD8^+^TILs and other cell types in HCC and ICC

**Fig. 6:**
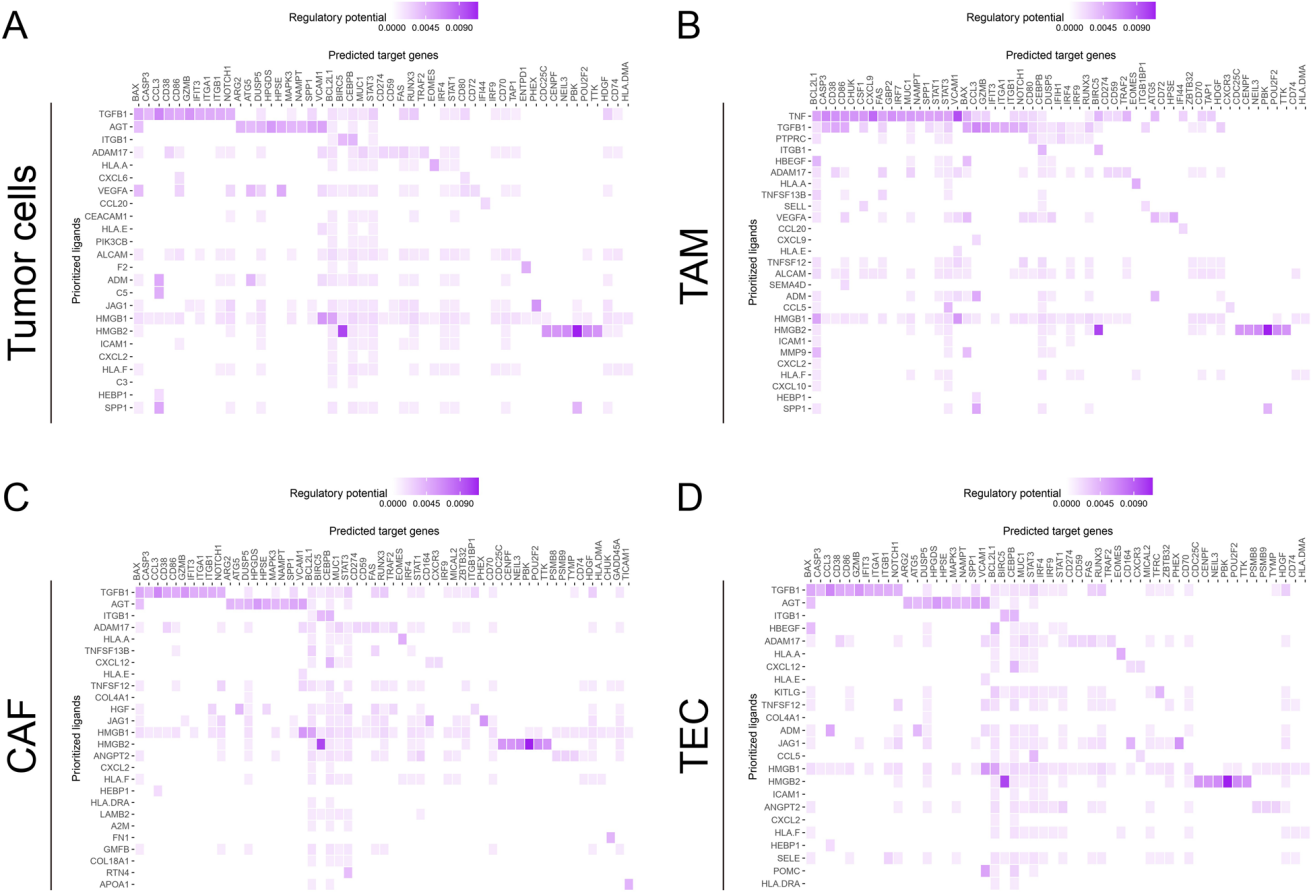
Cell–cell communication between CD8^+^TILs and other cell types in HCC and ICC. Heatmaps showed the regulatory potential of the prioritized ligands in tumor cells, TAMs, CAFs, and TECs that interact with signature genes of CD8^+^CD103^+^T cells

To study the regulation of CD8^+^CD103^+^TILs by other cells in the TME, scRNA-seq data of CD8^+^CD103^+^TILs in HCC and ICC were collected from GEO datasets (GSE: 125449) [7] according to the standard process of the Seurat package and UMAP analysis. To predict which signaling pathways were the most closely linked to CD8^+^CD103^+^TILs in TME of liver cancer, we adopted NicheNetr to explore tumor cells, tumor-associated macrophages (TAMs), cancer-associated fibroblasts (CAFs), and tumor endothelial cells (TECs) [35]. Single-cell analysis of HCC and ICC revealed potential ligand-receptor interactions. In CD8^+^CD103^+^TILs, several genes, such as *BAX*, *CASP3*, *CCL3*, *CD38*, *CD86*, *GZMB*, *IFIT3*, *ITGB1*, and *NOTCH1*, were predicted to have potential interactions with *TGFB1* on these TME-related cells. In addition, the engagement of TCR with TGF-β was involved in the *ITGAE* expression and the differentiation of CD8^+^CD103^+^T_RM_ cells. In particular, higher levels of *HMGB2* and *BIRC5*, the G2/M marker genes, were associated with encoding products linked to the cell cycle and proliferation of CD8^+^CD103^+^T_RM_ cells (Fig. 6A to D).

## Discussion

The dynamics of CD8^+^T-cell-mediated immune surveillance play an essential role in the continuous resistance and the outcome of intracellular infections and cancer [36]. It has been suggested that in the tumor model of transplantable cutaneous melanoma, the CD8^+^T_RM_ cells can promote a regional and durable melanoma-immune equilibrium within the skin [24]. Notably, the tissue-resident CD103^+^CD8^+^T cells in the TME are found to upregulate the inhibitory checkpoints, such as PD-1, CTLA-4, TIM-3, LAG3, TIGIT, and CD39. Therefore, these resident CD8^+^T cells are also considered tumor antigen-reactive T cells [37–39]. Furthermore, tissue-resident CD103^+^T cells are increased and respond to anti-PD-1 therapies in early-phase clinic trials [28]. Many retrospective studies have shown that tissue-resident CD103^+^CD8^+^TILs can be quantified as a significant predictor for the patient’s survival in many human solid tumors [37].

Our present study evaluated the expression of CD103 and immunolocalization of tissue-resident CD8^+^T cells in HCC and ICC patients using mIHC. HCC tissues exhibited a higher percentage of infiltrating CD8^+^T cells and CD103^+^ immune cells compared with the adjacent normal tissues. However, the percentage of CD103^+^CD8^+^T cells did not reveal any differences between HCC/ICC tissues and normal tissues. Some studies have demonstrated that CD103^+^CD8^+^T_RM_ cells represent highly activated T-cell subsets and provide tumor reactivity [40, 41]. We next evaluated the expressions of CD8 and CD103 to identify the clinical outcomes of liver cancer. Our results indicated that the intensity of infiltrating CD8^+^T cells had a prognostic value in human HCC and ICC. Furthermore, the HCC patients with CD8^high^CD103^high^ expression were associated with a better OS.

Moreover, the ICC patients expressing CD8^low^CD103^low^ T cells had a lower OS rate than the ICC patients with CD8^low^CD103^high^ and CD8^high^CD103^high^ expression. There was an improvement in OS regarding the patients with CD8^low^CD103^high^ and CD8^high^CD103^high^ expression in HCC or ICC, which might be related to the spatial heterogeneity of TILs in the immune microenvironment of HCC and ICC [42]. It was confirmed that in human HCC and ICC, the abundance of tumor-infiltrating CD8^+^T cells and CD103^+^CD8^+^T cells could be a prognostic predictor for patients’ OS. Furthermore, the percentages of CD103^+^CD8^+^TILs were also linked to the OS of HCC and ICC. Therefore, CD103 might be targeted for enhancing tumor immunity of CD8^+^T cells, and the infiltration of CD103^+^CD8^+^T cells in the liver TME might be considered as a biomarker to predict a better prognosis of the patients. The correlations between the numbers of CD8^+^TILs, CD103^+^ immune cells, and tissue-resident CD103^+^CD8^+^T cells and clinical parameters in HCC and ICC were investigated. We found that more CD8^+^T cells expressed CD103 in HCC and ICC tissues, which was negatively associated with the advanced pathological stage.

The tissue-resident CD103^+^CD8^+^T cells show their importance in anti-tumor immunity [43]. Further analysis is required to understand more immune checkpoint genes closely related to immune cell infiltration. The scRNA-seq data of CD3^+^TILs from human lung cancer reveal that the CD8^+^T-LAYN (PD-1^high^CTLA-4^high^) is the predominant subpopulation of CD103^high^ CD8^+^T cells, and these cells are the major subset in response to anti-PD-1 therapy [34]. Moreover, the patients with higher infiltration of tissue-resident CD103^+^CD8^+^T cells will receive greater survival-related benefits from anti-PD-1 therapy [37, 38]. In our present study, we also reanalyzed the published scRNA-seq data of human HCC and ICC. We found that the biological functions of CD103^+^CD8^+^TILs in human HCC or ICC tissues were highly consistent with those cells in human lung cancer [14, 17, 34]. Besides, some other inhibitory immune checkpoint molecules, such as *LAG3*, *TIGIT*, and *HAVCR2*, were also highly expressed in CD103^+^CD8^+^TILs in human HCC or ICC tissues, suggesting that those CD103^+^CD8^+^TILs were also the vital population in response to the immune checkpoint blockade therapy targeting LAG3, TIGIT or TIM3, or even in combination with anti-PD-1. In addition, based on the cell–cell communication analysis between CD103^+^CD8^+^TILs and other types of cells in human HCC or ICC tissues, some other cellular components played essential roles in regulating the biological functions of CD103^+^CD8^+^TILs. For example, various cell-derived TGF-β, and TAM-derived TNF-α, contributed to the formation of tissue-resident CD103^+^CD8^+^TILs in the TME. Moreover, the tumor cell-derived CXCL6, CXCL2, and CCL20, TAM-derived CXCL9/CXCL10, and the stromal cell-derived CXCL12 also contributed to the accumulation of tissue-resident CD103^+^CD8^+^TILs in the TME. Therefore, future clinical treatment targeting these molecules and cells may improve the effectiveness of the CD103^+^CD8^+^T-cell-mediated anti-tumor response in the TME.

In addition, there are also some limitations of our present study. First, the sample sizes of both HCC and ICC tissues need to be further extended and may improve the prognostic evaluation of tissue-resident CD103^+^CD8^+^T cells in these two malignancies. Second, due to the limited fluorescence channels could be selected in the multicolor immunohistochemistry assay, it would be better if we can add more functional markers to evaluate the anti-tumor response of tissue-resident CD103^+^CD8^+^T cells in the TME of both HCC and ICC. Third, due to the limitation of detailed therapeutic information of all the HCC and ICC patients involved in the present study, it is of great importance to evaluate the predictive value of tissue-resident CD103^+^CD8^+^T cells for therapeutic efficacy of target therapy or immunotherapy against HCC or ICC. Resolving these three limitations would help the clinical application of the examination of tissue-resident CD103^+^CD8^+^T cells in HCC or ICC tissues for the prediction of therapeutic efficacy and the understanding of immune regulation mechanism of these two malignancies.

Taken together, our present study indicated the prognostic values of infiltration intensity of tissue-resident CD103^+^CD8^+^TILs in human HCC and ICC. Moreover, we also revealed that the tissue-resident CD103^+^CD8^+^T cells were enriched in genes associated with T-cell activation, proliferation, cytokine function, and even T-cell exhaustion by using single-cell transcriptomics analysis.

## Conclusion

The CD103^+^ tumor-specific T cells signified an important prognostic marker with improved OS, and the evaluation of the tissue-resident CD103^+^CD8^+^T cells might help assess the status of TME in HCC and ICC.

## Supplementary Information

**Additional file 1:** **Supplementary Fig. 1.** A and D. Numbers of CD8+T cells between adjacent normal tissues and tumor tissues of HCC and ICC are shown by the violin plot, respectively. B and E. Numbers of CD103+ immune cells between adjacent normal tissues and tumor tissues of HCC and ICC are shown by the violin plot, respectively. B and E. Numbers of CD103+CD8+T cells between adjacent normal tissues and tumor tissues of HCC and ICC are shown by the violin plot, respectively. *P<0.05, **P< 0.01.

## Data Availability

All data generated or analyzed during this study are included in this published article.
