# Supplementary figures and images for: Prognostic values of tissue-resident CD8+T cells in human hepatocellular carcinoma and intrahepatic cholangiocarcinoma
Source: World J Surg Oncol. 2023 Apr 6;21:124. doi: 10.1186/s12957-023-03009-6 (PMC10077621; doi:10.1186/s12957-023-03009-6)

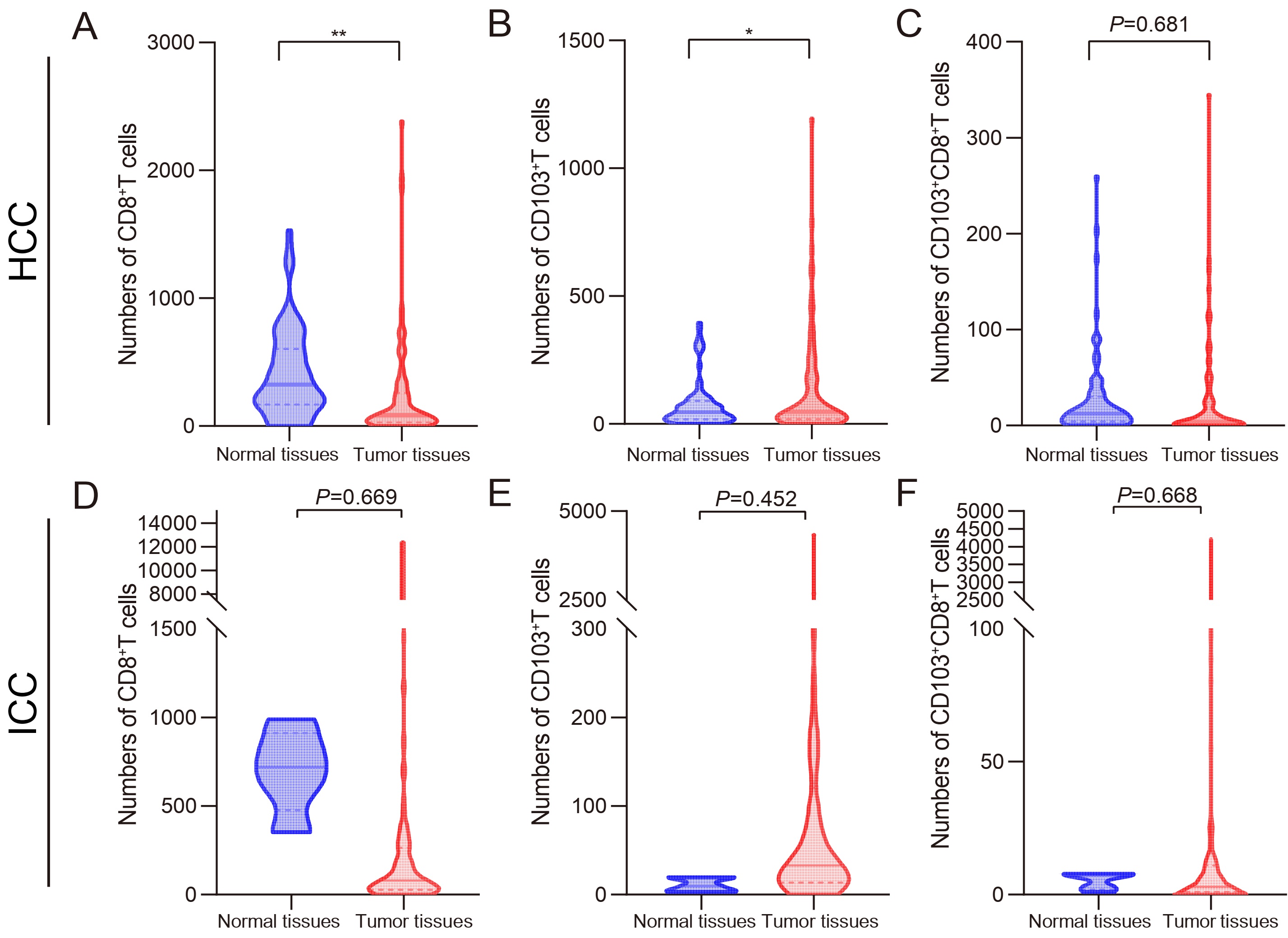

Supplement: Supplementary file 1 — Additional file 1: Supplementary Fig. 1. A and D. Numbers of CD8+T cells between adjacent normal tissues and tumor tissues of HCC and ICC are shown by the violin plot, respectively. B and E. Numbers of CD103+ immune cells between adjacent normal tissues and tumor tissues of HCC and ICC are shown by the violin plot, respectively. B and E. Numbers of CD103+CD8+T cells between adjacent normal tissues and tumor tissues of HCC and ICC are shown by the violin plot, respectively. *P<0.05, **P< 0.01. [file 12957_2023_3009_MOESM1_ESM.jpg]
